# Synthesis of Magnetic Adsorbents Based Carbon Highly Efficient and Stable for Use in the Removal of Pb(II) and Cd(II) in Aqueous Solution

**DOI:** 10.3390/ma14206134

**Published:** 2021-10-15

**Authors:** Safa Benjedim, Luis A. Romero-Cano, Hesham Hamad, Esther Bailón-García, Václav Slovák, Francisco Carrasco-Marín, Agustín F. Pérez-Cadenas

**Affiliations:** 1 Carbon Materials Research Group, Department of Inorganic Chemistry, Faculty of Sciences, University of Granada, Av. Fuente Nueva s/n., 18071 Granada, Spain; safabenj@correo.ugr.es (S.B.); heshamaterials@hotmail.com (H.H.); estherbg@ugr.es (E.B.-G.); fmarin@ugr.es (F.C.-M.); 2 Grupo de Investigación en Materiales y Fenómenos de Superficie, Departamento de Ciencias Biotecnológicas y Ambientales, Universidad Autónoma de Guadalajara, Av. Patria 1201, Zapopan 45129, Mexico; 3 Fabrication Technology Research Department, Advanced Technology and New Materials Research Institute (ATNMRI), City of Scientific Research and Technology Application (SRTA-City), Alexandria 21934, Egypt; 4 Department of Chemistry, Faculty of Science, University of Ostrava, 30, dubna 22, 702 00 Ostrava, Czech Republic; vaclav.slovak@osu.cz

**Keywords:** magnetic adsorbents, argan shells, adsorption, wastewater treatment, metals removal

## Abstract

In this study, two alternative synthesis routes for magnetic adsorbents were evaluated to remove Pb(II) and Cd(II) in an aqueous solution. First, activated carbon was prepared from argan shells (*C*). One portion was doped with magnetite (*Fe*_3_*O*_4_*+C*) and the other with cobalt ferrite (*CoFe*_2_*O*_4_*+C*). Characterization studies showed that *C* has a high surface area (1635 m^2^ g^−1^) due to the development of microporosity. For *Fe*_3_*O*_4_*+C* the magnetic particles were nano-sized and penetrated the material’s texture, saturating the micropores. In contrast, *CoFe*_2_*O*_4_*+C* conserves the mesoporosity developed because most of the cobalt ferrite particles adhered to the exposed surface of the material. The adsorption capacity for Pb(II) was 389 mg g^−1^ (1.88 mmol g^−1^) and 249 mg g^−1^ (1.20 mmol g^−1^); while for Cd(II) was 269 mg g^−1^ (2.39 mmol g^−1^) and 264 mg g^−1^ (2.35 mmol g^−1^) for the *Fe*_3_*O*_4_*+C* and *CoFe*_2_*O*_4_*+C*, respectively. The predominant adsorption mechanism is the interaction between -FeOH groups with the cations in the solution, which are the main reason these adsorption capacities remain high in repeated adsorption cycles after regeneration with HNO_3_. The results obtained are superior to studies previously reported in the literature, making these new materials a promising alternative for large-scale wastewater treatment processes using batch-type reactors.

## 1. Introduction

Lead and cadmium are highly toxic metals evolved to the environment due to anthropogenic activities such as metal finishing, electroplating, plastics, pigments, and mining industries [1]. Exposure to these metals can cause severe damage to the human body ranging from kidney damage, prostate damage, bone problems, and even neurological disorders [2]. Various methods have been studied that allow Cd(II) and Pb(II) elimination from bodies and water effluents. These methods range from precipitation, flocculation, electrochemical treatment, ion exchange, and membrane filtration processes [3,4,5]. These methods show very good results, but they are costly and, in most cases, not effective at low concentrations of the metal. The use of these processes generates high volumes of sludge, which is inefficient because they do not solve the environmental problem in its entirety. For this reason, the adsorption process has been the most widely used method, as it avoids all of the above problems [6].

In this regard, different types of carbon materials have been studied as adsorbent materials for the removal of Pb(II) and Cd(II), highlighting activated carbons [7,8,9], carbon nanotubes [10,11,12], graphene [13,14], fullerenes [15] and magnetic graphene oxides [16]. Although carbon materials are the preferred adsorbent material, it has shown some disadvantages when used in large-scale processes. The biggest of them is that when scaling up the process, packed fixed-bed columns must be used, which present mass transfer limitations resulting in a drastic decrease in the adsorption capacity of the material. An innovative strategy to solve this problem is the use of advanced carbon materials, such as magnetic carbons, which due to their properties, would be feasible to use in batch reactors to large-scale [17]. The mechanical agitation in these reactors favors the mass transfer phenomena so that the adsorption capacity does not decline. Finally, using an external magnet, it would be possible to separate the solid quickly and effectively.

At the moment, there are different methodologies reported to synthesize this type of material [18,19,20,21,22,23]. Most of them have the disadvantage that the adsorbent material obtained is not stable and is inefficient in repeated cycles of use, limiting its large-scale applications. For this reason, the current challenge and the aim of this study consists of obtaining magnetic carbons that retain their properties in repeated adsorption-desorption cycles, so the present study shows a new alternative for preparing these materials. It is expected that the incorporation of magnetic nanoparticles to an activated carbon will improve the adsorption capacities of the carbon material, making it possible to use them in repeated adsorption-desorption cycles. For this reason, in the first stage, activated carbon with a high surface area was synthesized, for which argan shells were used as a precursor material. In the second step, the dispersion of magnetite and cobalt ferrite particles on their surface was studied. To obtain information of the materials, their physicochemical characterization was carried out as well as adsorption studies of Pb(II) and Cd(II) to evaluate the adsorption mechanism and project their use in large-scale wastewater treatment processes.

## 2. Materials and Methods

### 2.1. Preparation of Activated Carbon Impregnated with Magnetite and Cobalt Ferrite: Magnetic Adsorbents

Magnetic activated carbons were prepared according to a co-impregnation method. Activated carbon support (*C*) was prepared using the methodology proposed by Bejedim et al., (2020) [24], and then the magnetite nanoparticles were prepared via the modified chemical co-precipitation method [25]. Briefly, 1 g of FeCl_2_ and 2 g of FeCl_3_ (molar relation of Fe(III)/Fe(II) ≈ 2:1; in solution) was dissolved in 200 mL of distilled water under N_2_ flow with vigorous stirring at 30 °C; in the second step, 1 g of *C* was added, after that 10 mL of ammonium hydroxide (25%) was put into the mixture producing a color change from orange to dark brown. The solid was recuperated and washed with deionized water, and ethanol and dried at 60 °C for 24 h. Finally, a heat treatment was carried out at 500 °C under a nitrogen atmosphere for 2 h with a heating ramp of 2 °C min^−1^. This sample was labeled *Fe_3_O_4_+C.*

The same procedure was carried out to prepare an activated carbon impregnated with cobalt ferrite nanoparticles. In this case, 1 g of CoCl_2_ was used with 2 g of FeCl_3_ dissolved in 100 mL of distilled water at 95 °C. After that, 80 mL of ammonium hydroxide (1 M) were added. Finally, the solid was filtered, washed, dried, and heat-treated. The resulting sample was labeled *CoFe_2_O_4_+C.*

### 2.2. Physicochemical Characterization of the Magnetic Adsorbents

The textural properties (specific surface areas, and size distribution of porosity) of the magnetic adsorbents were studied by the physisorption of N_2_ at −196 °C, for which an Autosorb1 from Quantachrome Inc (Anton Paar QuantaTec, Boynton Beach, FL, USA) equipment was used. 

The morphology of the magnetic adsorbents was studied through a scanning electron microscopy (SEM-EDX), using a Zeiss Leo 1530 Gemini Field Emission scanning electron microscope (Zeiss, Jena, Germany), and transmission electron microscopy (TEM) using a LIBRA 120 PLUS (Carl Zeiss SMT, Jena, Germany) microscope. 

The thermal stability of the materials was evaluated in a thermogravimetric analyzer, Mettler TA 400 (Mettler-Toledo International Inc, Greifensee, Switzerland) The experimental conditions were as follows: air atmosphere, (100 mL min^−1^); initial mass, 100 mg; temperature range, 25–900 °C; and a heating rate of 10 °C min^−1^. 

The functional groups present in the synthesized materials were studied though a Fourier transform infrared spectroscopy (4000–400 cm^−1^), using a Nicolet 6700 FTIR spectrometer (Thermo Fisher Scientific, Waltham, MA, USA). To characterize the chemical surface of the magnetic adsorbents, the X-ray photoemission spectroscopy (XPS) was used; the equipment used was a Kratos Axis Ultra-DLD X-ray photoelectron spectrometer (Kratos Analytical Ltd., Kyoto, Japan). The experimental conditions were a monochromatic X-ray source Al Kα (1486.71 eV); maintained pressure condition of 6 × 10^−10^ Torr in the analytical chamber; wide-scan spectra, 0–1100 eV; step energy, 80 eV; and step size, 1 eV. The high-resolution scans were performed for the C_1s_, O_1s_, Fe_2p_ and Co_2p_ regions (experimental conditions: step energy 40 eV, step size 0.05 eV). 

The zeta Potential was measured by first dispersing *Fe_3_O_4_+C* and *CoFe_2_O_4_+C* in 1 mmol L^−1^ NaCl solution by sonication in order to obtain the supernatant with Zetasizer Nano ZS (Malvern Panalytical, Malvern, England).

Finally, the magnetic properties of the materials were studied by a vibrating sample magnetometer (VSM) using the equipment, VersaLab, Quantum (Quantum Design, San Diego, CA, USA), at room temperature.

### 2.3. Adsorption Studies of Metal Cations in Aqueous Solutions

The removal of lead and cadmium cations in aqueous solutions was studied using *C*, *Fe*_3_*O*_4_*+C* and *CoFe*_2_*O*_4_*+C* materials. Pb(II) solutions were prepared using Pb(NO_3_)_2_ in distilled water (200 to 3600 mg L^−1^). On the other hand, Cd(II) solutions were prepared using Cd(NO_3_)_2_ in distilled water (100 to 1000 mg L^−1^). The experimental conditions were: 0.1 g of adsorbent, 50 mL of the metal solution, agitation of 180 rpm at 25 °C for at least 24 h (time required to reach equilibrium). The experiments were carried out using a buffer solution with a constant pH (pH = 5) prepared from 0.1 M acetic acid to 0.2 M sodium acetate.

The adsorbed amount of metal ions was calculated by Equation (1):

(1) $$q_{e}=\left(C_{0}-C_{e}\right)\frac{V}{W}$$

where *C*_0_ (mg L^−1^) and *C_e_* (mg L^−1^) are the initial and equilibrium concentrations, *V* (L) is the volume of solution, and *W* (g) is the mass of material employed.

The concentration of metals in the solution was measured using a Varian atomic absorption spectrophotometer (Varian AA240FS Varian Inc., Palo Alto, Santa Clara, CA, USA) at 283.3 and 228.8 nm for Pb(II) and Cd(II) determination, respectively, using an acetylene-air flame. 

#### 2.3.1. Adsorption Kinetics

The adsorption kinetic studies were conducted using an initial concentration of 500 mg L^−1^ at pH 5 using a buffered solution prepared with 0.1 mol L^−1^ of acetic acid and 0.2 mol L^−1^ of sodium acetate.

In order to obtain fundamental information that allows for describing the effect of time on the adsorption of Pb(II) and Cd(II) on the magnetic adsorbents, the adjustment of the experimental data to the mathematical models described in Table 1 was tested.

#### 2.3.2. Adsorption Equilibrium

Once the experimental data of *C_e_* vs. *q_e_* was obtained, its adjustment to the mathematical models proposed by Langmuir and Freundlich was tested (Table 1). Finally, the effect of pH on adsorption capacity was studied in the pH range of 2 to 8. For this purpose, the pH of aqueous solutions Pb(II) and Cd(II) was changed to the needed value by addition of HCl or NaOH solutions.

#### 2.3.3. Reuse of the Materials (Adsorption—Desorption Studies)

Desorption and reusability of adsorbent materials were studied employing HNO_3_ (0.05 M) as a desorbing solution. The experiments were carried out employing 0.1 g of saturated adsorbent in 20 mL of HNO_3_, and were agitated at 180 rpm, at 25 °C. After elution, the concentration of metals in the desorbing solution was analyzed by atomic absorption, and the saturated adsorbent was washed with ethanol and dried at room temperature. After that, the adsorbent was reused for cations adsorption in four successive cycles at pH = 6.

### 2.4. Statistical Analysis

All the experiments were carried out in duplicate. The data presented in the figures correspond to the average value. The analysis of variance (ANOVA) was performed for the removal of Pb(II) and Cd(II) concentration as a function of contact time, initial metal concentrations, pH regimes, and reuse cycles to determine significant differences using the STATISTICA 10.0 software (StatSoft, Inc., Tulsa, OK, USA). 

Mathematical models were applied and adjustments of the data to the nonlinear equations were carried out using as an estimation method, the algorithm of Levenberg–Marquardt in the STATISTICA software. The Levenberg–Marquardt Algorithm (nonlinear least squares) is an efficient method for estimating the parameters of nonlinear regression models, when using the least-squares loss function. The input data for the STATISTICA software are the amount of adsorbate per unit amount adsorbent (q) vs. time (kinetic models) and q vs. concentration of adsorbate at equilibrium (isotherms models). The objective function used in correlating the data was $\left|q_{exp}-q_{pred}\right|/q_{exp}$ where superscripts *exp* and *pred* represents the experimental and calculated/predicted values, respectively. The correlating ability of the various models was compared in terms of the correlation coefficient (R).

## 3. Results and Discussion

### 3.1. Physicochemical Characterization of the Magnetic Adsorbents

**Figure 1:**
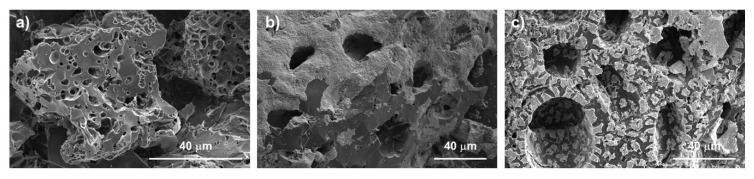
SEM images. Samples: (**a**) *C*, (**b**) *Fe*_3_*O*_4_*+C*, and (**c**) *CoFe*_2_*O*_4_*+C*.

Figure 1 shows the adsorption-desorption isotherms of N_2_ (Supplementary Materials, Figure S1a) and the pore size distribution (Figure S1b) obtained for all materials. The results obtained are shown in Table 2. For activated carbon used as a support (*C*), a value of W_0_ 0.612 cm^3^ g^−1^ is observed, evidencing the high degree of activation of the material. This can be clearly seen in the BET surface area of the material (S_BET_), which is 1635 m^2^ g^−1^. However, once the material is impregnated with magnetite and cobalt ferrite particles, there are significant changes in the material’s texture. First, the BET area decreases to 394 and 359 m^2^ g^−1^, respectively. Although the decrease was to be expected, in the pore size distribution there are significant differences between both magnetic carbons. For the *Fe*_3_*O*_4_*+C* material, the pore size distribution is in a range of 10 to 100 nm, while for *CoFe*_2_*O*_4_*+C,* the range is less than 10 nm, indicating that the precursor used to add magnetic properties to the material directly influences the texture of the final material.

This information can be corroborated with the morphology studies of the materials. Figure 1 shows the SEM images for each of the materials. For the *C* sample (Figure 1a) the channels developed due to activation with KOH are clearly appreciated. However, for the *Fe*_3_*O*_4_*+C* material (Figure 1b) these channels become narrower due to impregnation with magnetite nanoparticles. The modification studied provides particle sizes in the order of nanometers, so that these can penetrate the texture of the carbon material saturating the microporosity. This was shown in the pore size distribution. On the other hand, in the *CoFe*_2_*O*_4_*+C* material (Figure 1c) it can be seen that modifications grant larger cobalt ferrite particles. Most of them are anchored on the outermost surface of the material, for this reason, it preserves the initial and contracts the more exposed channels. The images obtained are shown in Figure 1, which confirms that the nanoparticles were able to impregnate the entire internal texture of activated carbon, while cobalt ferrite particles remain less dispersed, mainly concentrating on the surface of the material.

Figure S2 shows the TEM images of each of the materials obtained; it can be observed that for the case of the *Fe*_3_*O*_4_*+C* sample, nanometric particles were obtained with an average size of 36 nm. On the other hand, for the material *CoFe*_2_*O*_4_*+C,* the size was 14 nm. This can be attributed to the fact that during the synthesis of the materials, agglomerates of magnetite nanoparticles are generated, increasing the volume and size of the nanoparticles; otherwise, the cobalt ferrite nanoparticles disperse correctly on the carbon material.

The magnetite and cobalt ferrite contents in the magnetic carbons were evaluated through thermogravimetric analysis. The TG curves are presented in Figure S3, from which the magnetite and cobalt ferrite contents supported on *C* were determined to be 56.6 and 51.8 wt%, respectively. Considering that the compounds present in the magnetite are mostly Fe_3_O_4_, the Fe content is calculated to be 40.9 wt%. On the other hand, for cobalt ferrite, the predominant species is CoFe_2_O_4,_ so that the Fe and Co contents are 28.5 and 15.0%, respectively.

**Figure 2:**
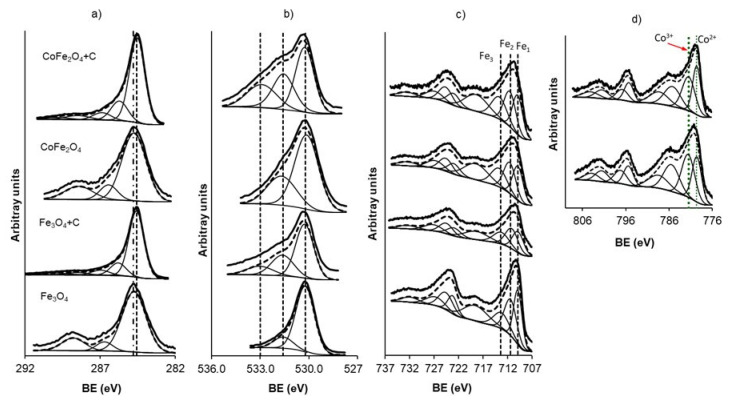
Deconvolution of the high-resolution XP spectra into the main species: (**a**) C_1s_; (**b**) O_1s_; (**c**) Fe_2p_ and (**d**) Co_2p_. Samples: Fe_3_O_4_ and *Fe*_3_*O*_4_*+C* (**down**), and CoFe_2_O_4_ and *CoFe*_2_*O*_4_*+C* (**up**).

The results of the XP spectroscopy studies are presented in Figure 2 and Table 3. The deconvolution of the C_1s_ spectrum, for magnetic carbon samples, shows five peaks (Figure 2a), which correspond to bonds: C=C (peak at 284.6 eV), C-O (peak at 285.8 eV), C=O (peak at 287.0 eV), O=C-OR (peak at 288.5 eV) and ${\mathrm{CO}}_{3}^{—}$ species (peak at 290.0 eV) [24,31]. For metal oxides the C_1s_ spectrum is shifted to a higher binding energy (BE), C=C (peak at 284.8 eV) which corresponds to adventitious carbon [32].

This analysis agrees with the deconvolution of the O_1s_ spectra, as shown in Figure 2b, which shows three peaks for magnetic carbon samples. The first of them, 530.1 eV, corresponds to oxygen bonded to +2 and +3 cations [33]. The peak centered 531.6 eV corresponds to the C=O bond and the finally the peak at 533.0 eV is related to the C-O bond [24,34,35,36]. In the case of metal oxides, only two peaks are detected in O bonded to metal cations and C=O is present in adventitious carbon. 

The XP spectra for the Fe_2p_ region are shown in Figure 2c. The mathematical deconvolution of the spectra shows the typical doublet of iron, consisting of two signals (712 and 725 eV); these correspond to the 2p_3/2_ and 2p_1/2_ contributions, respectively [37]. The position and energy gap coincide to those observed for the FeO(OH) phases [38]. Nevertheless, the deconvolution of the spectrum was performed in three double peaks being the Fe_2p_3/2 BE at 709.9 ± 0.2, 711.2 ± 0.3 and 713.2 ± 0.2 eV attributed to Fe^2+^ placed in octahedral holes (Fe_1_), Fe^3+^ placed in octahedral holes (Fe_2_), and Fe^3+^ placed in tetrahedral holes (Fe_3_), respectively. Therefore iron atoms are taking part of the chemical species type $M_{x}^{2+}M_{1-x}^{3+}[Fe_{y}^{2+}Fe_{1-y}^{3+}]O_{4}$ [39] being M = Co or simply magnetite.

Finally, Figure 2d shows the deconvoluted spectra of Co_2p_ for the CoFe_2_O_4_+C sample. The spectrum was deconvoluted into four peaks centered at 779.5 ± 0.2, 781.6 ± 0.2, 785.4 ± 0.2 and 788.4 ± 0.2 eV. The peaks at 779.5 ± 0.1 are associated with Co^2+^ placed in tetrahedral holes, and the peaks at 781.6 ± 0.2 are associated with Co^2+^ placed in octahedral holes [39]. This means that metals situated on the surface of the samples are taking part of chemical species type $(Co_{x}^{2+}Fe_{y}^{3+})[Fe_{z}^{2+}Fe_{1-y}^{3+}Co_{1-x}^{2+}]O_{4}$, where cations in parenthesis are situated in tetrahedral positions while cations in brackets are situated in octahedral positions. The peaks around 785.4 ± 0.2 and 788.4 ± 0.2 eV correspond to 2p_3/2_ satellite signals.

Therefore, the XPS study clearly indicates the presence of magnetic phases, not ruling out the coexistence of surface complexes of the Fe-OH type.

**Figure 3:**
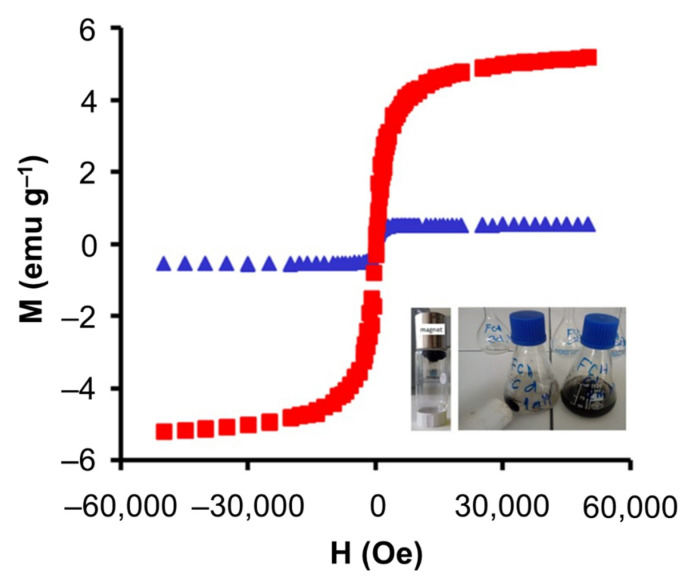
Magnetization curves of ■ *Fe*_3_*O*_4_*+C*, and ▲ *CoFe*_2_*O*_4_*+C*. Inset is a photograph of the material being attracted by an outer magnet.

Figure 3 shows the magnetization curves of magnetic carbons. The corresponding magnetization saturation values for *Fe*_3_*O*_4_*+C* and *CoFe*_2_*O*_4_*+C* were 5.19 and 0.54 emu g^−1^, respectively. This property of the materials is advantageous to carry out wastewater treatment processes in batch mode. A significant disadvantage that adsorption processes present today is related to their industrial scalability. A strategy to achieve this is to use fixed-bed columns; however, it was shown that this mode of operation drastically decreases the adsorption capacity of the materials due to mass transfer limitations. The magnetic condition of these new materials opens the possibility of studying adsorption processes in batch mode on a large scale; this condition allows for preserving the adsorption capacity of the materials since the condition of agitation can be preserved and favor the surface phenomenon, and later efficiently separate the material using an outer magnet. As evidenced by the image in the box within Figure 3.

Figure S4 and Table 2 show the zeta potentials of the materials. The isoelectric point of the *C* material was pH 8.02, the basic condition of the material is attributable to the type of activating agent used (KOH). Once the impregnation is carried out, the isoelectric point of the material changes to pH = 3.5 and pH = 3.4 for the *Fe*_3_*O*_4_*+C* and *CoFe*_2_*O*_4_*+C* materials, respectively. It confirms that the modifications were carried out successfully since the acidic properties of the material due to the oxides and metallic species that are now present on the surface of the C support are evident.

### 3.2. Adsorption Studies

**Figure 4:**
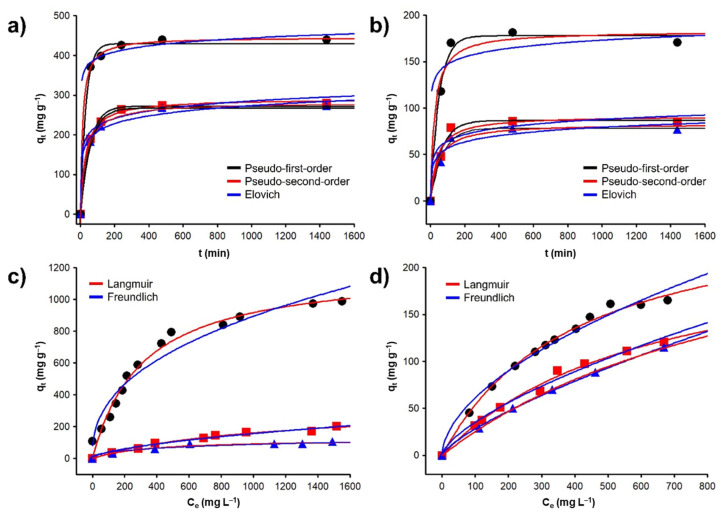
Effect of time on the adsorption of (**a**) Pb(II) and (**b**) Cd(II). Adsorption isotherms of (**c**) Pb(II) and (**d**) Cd(II) onto ● *C*, ■ *Fe*_3_*O*_4_*+C*, and ▲ *CoFe*_2_*O*_4_*+C*.

The results obtained from the adsorption kinetics are shown in Figure 4a,b. The adsorption of the metals increases significantly (*p* < 0.01) with the increment of the contact time. It is observed that for all the materials tested, the adsorption process is slow, reaching 97% of the adsorption after 240 min (4 h) this time was sufficient to achieve the equilibrium state and the additional increment of the contact time did not change significantly (*p* > 0.05). The *C* sample shows the highest adsorbed amount for both metals (at used conditions), 439 mg g^−1^ (2.12 mmol g^−1^) for Pb(II) and 178 mg g^−1^ (1.58 mmol g^−1^) for Cd(II); this effect can be described due to the adsorption mechanism involved. To obtain information in this regard, the kinetic models of the pseudo-first-order, pseudo-second-order, Elovich, and intraparticle diffusion were evaluated. The kinetic constants obtained are shown in Table 4. Among them, the first two models were the ones that best represented the experimental data. When comparing the kinetic constants k_1_ and k_2_ of the materials, it is evident that the adsorption in *C* is faster, probably because the active sites are more available. Additionally, the adsorption kinetics of *C* is better represented by the pseudo-first-order model (R = 0.99), contrary to the *Fe*_3_*O*_4_*+C* and *CoFe*_2_*O*_4_*+C* materials whose experimental data can be described by the pseudo-second-order model (R = 0.99). Based on this information, it is proposed that a physisorption phenomenon is involved for the *C* material, while for magnetic carbons, the limiting step in adsorption is a chemical interaction between adsorbent and adsorbate.

The adsorption isotherms for Pb(II) and Cd(II) are shown in Figure 4c,d. The adsorption of the metals increased significantly (*p* < 0.01) with the increase of the initial concentration. The fit of the experimental data to the Langmuir and Freundlich isotherm models was tested. The constants obtained are shown in Table 5. For the case of the *C* material, an adsorption capacity of 989 mg g^−1^ (4.77 mmol g^−1^) for Pb(II) and 165 mg g^−1^ (1.47 mmol g^−1^) for Cd(II) was obtained. For all cases, the Langmuir isotherm model is the one that best represents the experimental data, indicating that the adsorption process is due to the formation of a monolayer of the adsorbate on the adsorbent surface, which is more evident in the *C* sample. Since the asymptotic value of the model is clearly marked, this can be described because the adsorption mechanism is a cation-π interaction between the graphitic planes of the material and the cation in solution [40], The aromatic carbon on the surfaces of the materials produces electron-rich π-systems that may donate π-electrons to interact with cations, which may act as π-acceptors because of electron deficiencies [41]; in such a way the larger the area surface of the material, the greater its adsorption capacity.

In the case of magnetic carbons, adsorption capacities are lower than those achieved by *C*. If the adsorption process on these materials were identical to that discussed above, the decrease in adsorption capacity should be in the same order that the specific surface area of the materials decreases due to the formation of the monolayer due to cation-π interactions, but it is clear that this does not happen. While this process may exist for *Fe*_3_*O*_4_*+C* and *CoFe*_2_*O*_4_*+C* materials, it is not the phenomenon that governs adsorption. Due to this, it is proposed that there is a second mechanism, similar to what has been previously reported [42,43,44,45].

**Figure 5:**
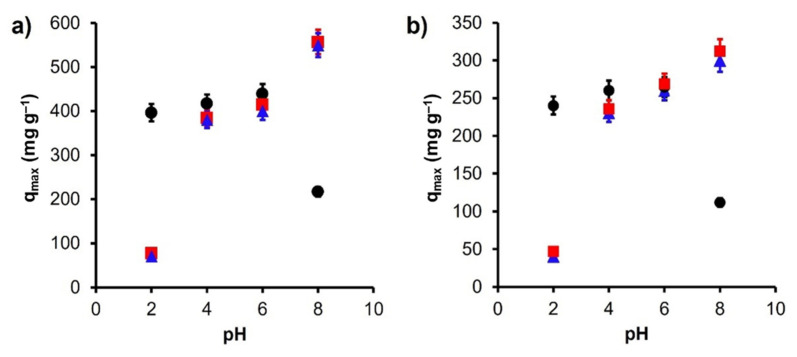
Effect of pH on adsorption of (**a**) Pb(II) and (**b**) Cd(II) onto ● *C*, ■ *Fe*_3_*O*_4_*+C*, and ▲ *CoFe*_2_*O*_4_*+C*.

In order to obtain more information on the adsorption mechanism, adsorption tests were carried out in a pH range between 2 and 8. The effect of pH significantly affected (*p* < 0.01) the adsorption of Pb(II) and Cd(II). The results obtained are shown in Figure 5. For the *C* material, it is observed that the adsorption capacity increases slightly with increasing pH; however, for basic pH ranges, the adsorption drops drastically. This effect can be explained due to the pH_pzc_ (8.02) of the material, which at a pH = 8.0 results in a neutral charge on the surface of the material, decreasing the charge density in the π-system, weakening the interaction with the cation in solution. On the other hand, a drastic increase is observed for magnetic carbons when pH increases in the solution; this effect can be explained similarly. Due to the impregnation of magnetite and cobalt ferrite, the pH_pzc_ of the original material was modified, decreasing to a value of 3.5; this modification allows the materials to be useful in a wide pH range, since only when the pH of the solution is less than 3.5, it restricts the adsorption of cations as a result of a repulsive force due to the positive surface charge of the materials. An important detail to highlight is that for pH values above 8 the cations begin to precipitate from the solution, so the optimal range for the adsorption process can be defined between 3.5 and 8.

**Figure 6:**
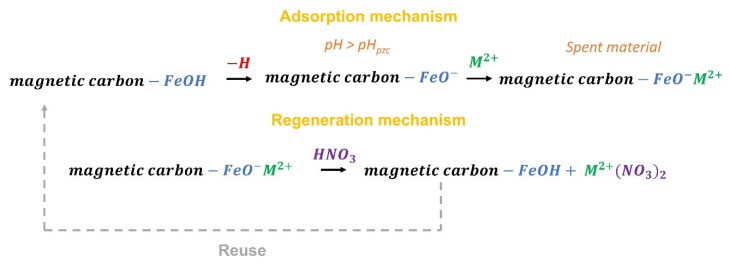
Adsorption and regeneration mechanism proposed for Cd(II) and Pb(II) onto magnetic carbons.

Consequently, the second adsorption mechanism present in magnetic carbons is consistent with the presence of -FeOH groups and its interaction with the metal ions in solution. It is very probable that the adsorption mechanism is associated with the interaction of metal ions with deprotonated -FeOH groups (at pH > pH_pzc_), which would provoke a negative charge on the surface of the materials attracting by electrostatic forces the cations in solution. This mechanism is detailed in Figure 6.

Although the *C* material has an extraordinary adsorption capacity in the first cycle, it begins to decline drastically in the following cycles (*p* > 0.05), losing 81% of its capacity for Pb(II) and 41% for Cd(II) in the fourth cycle. This effect can be attributed to the fact that there are adsorption sites that are available in the first cycles, which cannot be regenerated because they are inside the pores of the material, and due to the shape of the pore and size of the adsorbed cations, an obstruction phenomenon preventing its desorption occurs.

**Figure 7:**
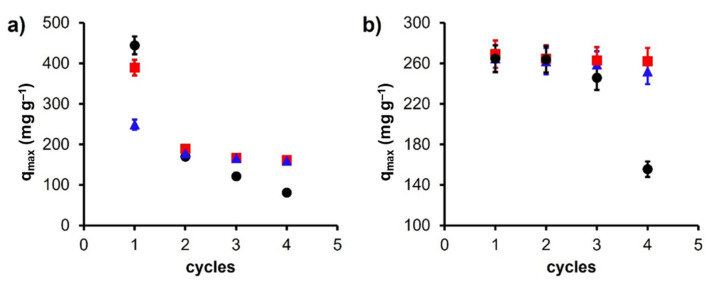
Adsorption capacity of regenerated materials for four consecutive adsorption-desorption cycles (pH 6) for (**a**) Pb(II) and (**b**) Cd(II). Samples: ● *C*, ■ *Fe*_3_*O*_4_*+C*, and ▲ *CoFe*_2_*O*_4_*+C*.

When analyzing the adsorption-desorption cycles in magnetic carbons (Figure 7), it is observed that the adsorption capacity does not decline in the same way after the first cycle (*p* < 0.01). In the case of Pb(II) adsorption at the fourth cycle, the capacity of the material drops by 58% for *Fe*_3_*O*_4_*+C* and 35% for *CoFe*_2_*O*_4_*+C*. This can be attributed to the adsorption mechanism discussed above. In the first adsorption cycle, the cations are adsorbed by two adsorption mechanisms: the interaction between -FeOH groups with the cation (principally) and by a cation-π interaction; due to the second mechanism, in the regeneration process, some cations are trapped in the texture of the material due to the clogging phenomena already discussed. Because of this, the drastic decrease in the capacity of the materials in the second adsorption cycle occurs (51% for *Fe*_3_*O*_4_*+C* and 28% for *CoFe_2_O_4_+C*). Once these sites are disabled for subsequent cycles, adsorption remains unchanged because adsorption is now only governed by the interactions with -FeOH groups. The regeneration of the adsorbent is possible through the incorporation of HNO_3_, which exchanges a proton with the cation adsorbed in the material, putting it back to its original state to be used in repeated adsorption-desorption cycles (Figure 6). A similar effect is observed for Cd(II) adsorption studies. In this case, the reduction in *C* capacity in the repeated cycles is clear. However, for magnetic carbons, it remains constant, showing that from the first cycle the predominant adsorption phenomenon is cation exchange.

Table 6 shows a comparison of the results obtained against studies recently published in the literature in which magnetic carbons prepared from agro-industrial waste are used. This highlights the materials prepared in this study, which have high adsorption capacities, showing their effectiveness in repeated adsorption cycles. This condition demonstrates the suitability of the materials for large-scale wastewater treatment processes, since the cost of regeneration is low, and may even not be necessary in continuous cycles due to their high adsorption capacities.

## 4. Conclusions

Magnetic carbons were prepared from argan shells and supporting magnetite and cobalt ferrite particles. The carbons impregnated with cobalt ferrite preserved the mesoporous texture of the support because the particles were mainly dispersed on the outermost surface of the material. The opposite was found for magnetite particles, which were introduced into the texture of the material. The adsorption of metals increased significantly (*p* < 0.01) with the increment of the contact time. The equilibrium state was achieved after 240 min and the increment of the contact time did not affect (*p* > 0.05) the adsorption capacity significantly. The adsorption of Pb(II) and Cd(II) increased significantly (*p* < 0.01) with the increase of the initial concentration. The effect of pH in the adsorption process was strongly affected (*p* < 0.01) because the predominant mechanism is due to the interaction between the -FeOH groups with the cation in the solution. Finally, the adsorption capacity of magnetic adsorbents does not decline significantly after the first cycle (*p* < 0.01). The results obtained are superior to the studies previously reported in the literature, making these materials a promising alternative for large-scale wastewater treatment processes using batch type reactors with mechanical stirring.

## Figures and Tables

**Table 1:** Mathematical models tested to describe the adsorption kinetics and equilibrium of Pb(II) and Cd(II) onto magnetic adsorbents.

Model	Equation	Parameters	Reference
Pseudo-first-order	$q_{t}=q_{e}\left(1-e^{-k_{1}t}\right)$	*q_e_* (mg g^−1^) and *q_t_* (mg g^−1^): the amounts of adsorbed adsorbate at equilibrium and at time *t*. *k*_1_ (min^−1^): rate constant of pseudo-first-order adsorption.	[26]
Pseudo-second-order	$q_{t}=\frac{1}{\frac{1}{k_{2}q_{e}{}^{2}}+\frac{t}{q_{e}}}$	*k*_2_ (g mg^−1^ min^−1^): equilibrium rate constant of pseudo-second-order adsorption.	[27]
Elovich	$q_{t}=\frac{2.3}{\alpha }\times log\left(1+\alpha \beta t\right)$	*α* (mg g^−1^ min^−1^): sorption rate. *β* (g mg^−1^): extent of surface coverage and activation energy for chemisorption.	[28]
Intraparticular diffusion	$q_{t}=k_{i}t^{1/2}$	*k_i_* (mg g^−1^ min^−1/2^): intraparticle diffusion rate constant.	[28]
Langmuir	$q_{e}=\frac{q_{max}KC_{e}}{1+KC_{e}}$	*q_max_* (mg g^−1^): adsorption capacity of the material. *K* (L mg g^−1^): Langmuir constant.	[29]
Freundlich	$q_{e}=K_{f}C_{e}{}^{1/n}$	*K_f_*: Freundlich constant. *1/n*: heterogeneity factor.	[30]

**Table 2:** Physicochemical characterization of materials.

Material	S_BET_	L_0_	W_0_	V_0.95_	V_meso_	pH_pzc_
	(m^2^ g^−1^)	(nm)	(cm^3^ g^−1^)	(cm^3^ g^−1^)	(cm^3^ g^−1^)	
*C*	1635	1.00	0.612	0.715	0.103	8.0
*Fe*_3_ *O*_4_ *+C*	394	1.04	0.158	0.209	0.051	3.5
*CoFe*_2_ *O*_4_ *+C*	359	1.22	0.144	0.239	0.095	3.4

**Table 3:** Binding energies (eV) of C_1s_, O_1s_, Fe_2p,_ and Co_2p_, regions of the carbon materials prepared from argan seed shell.

Sample	C_1s_	FWHM *	Peak	O_1s_	Peak	O		Sample	Fe_2p_		Fe		Co_2p_	Peak	Co		%Fe(II)	%Fe(III)	%Fe(III)	%Fe(III)
	eV	eV	%	eV	%	% (Mass)	% (Atomic)		eV		% (Mass)	% (Atomic)	eV	%	% (Mass)	% (Atomic)	Octa	Tetra	Octa	Tetra
*Fe_3_O_4_*	284.8	1.86	77	530.2	85	31.8	49.8	*Fe*_3_ *O*_4_	709.9	38.0	56.18	25.2	0.0				38.0	62.0	61.5	38.5
	286.7		8	531.6	15				711.4	38.1										
	288.8		15						713.3	23.8										
									718.6											
									723.2											
									724.6											
									726.7											
									731.9											
*Fe*_3_ *O*_4_ *+C*	284.6	1.18	72	530.3	59	16.4	15.9	*Fe*_3_ *O*_4_ *+C*	709.9	34.1	23.42	6.5	0.0				34.1	65.9	57.7	42.3
	285.8		14	531.6	27				711.2	38.1										
	287.0		7	533.0	13				713.3	27.9										
	288.5		4						718.4											
	290.0		3						722.9											
									724.5											
									727.1											
									732.4											
*CoFe_2_O_4_*	284.8	1.89	72	530.1	68	30.7	46.6	*CoFe*_2_ *O*_4_	709.9	35.3	33.26	14.5	779.6	44.7	21.03	8.7	35.3	64.7	56.3	43.7
	286.4		11	531.7	32				711.6	36.4			781.6	55.3						
	288.4		16						713.7	28.3			785.4							
									718.4				788.4							
									723.0				795.4							
									724.6				797.2							
									726.8				801.6							
									732.5				803.9							
*CoFe*_2_ *O*_4_ *+C*	284.6	1.21	69	530.3	44	16.2	14.8	*CoFe*_2_ *O*_4_ *+C*	709.9	34.6	10.76	2.8	779.6	47.8	7.34	1.8	34.6	65.4	55.1	44.9
	285.7		16	531.6	31				711.5	36.1			781.5	52.2						
	286.9		7	532.9	25				713.7	29.3			785.2							
	288.5		5						718.4				788.3							
	289.9		3						723.0				795.2							
									724.7				796.8							
									727.0				801.5							
									732.6				804.2							

* FWHM: full width at half maximum.

**Table 4:** Kinetic constants of the models tested for Pb(II) and Cd(II) adsorption onto magnetic adsorbents.

		q_e,exp_	Pseudo First Order			Pseudo Second Order			Elovich			Intraparticle Diffusion	
			k_1_	q_e, calc_	R	k_2_	q_e, calc_	R	α	β	R	k_i_	R
		(mg g^−1^)	(min^−1^)	(mg g^−1^)		(g mg^−1^ min^−1^)	(mg g^−1^)					(mg g^−1^ min^−1/2^)	
*C*	Pb(II)	439.0 ± 22.0	0.0314	429.5	0.997	0.00179	445.8	0.988	0.105	61 × 10^5^	0.997	17.14	0.000
*Fe*_3_ *O*_4_ *+C*	Pb(II)	280.0 ± 14.0	0.0185	272.4	0.997	0.00113	290.6	0.999	0.084	393.09	0.991	10.57	0.294
*CoFe*_2_ *O*_4_ *+C*	Pb(II)	272.0 ± 13.0	0.0174	267.4	0.997	0.00109	281.7	0.999	0.081	194.07	0.994	9.54	0.508
*C*	Cd(II)	177.9 ± 9.0	0.0196	178.0	0.996	0.00229	182.8	0.987	0.204	23 × 10^3^	0.972	4.57	0.000
*Fe*_3_ *O*_4_ *+C*	Cd(II)	85.3 ± 4.0	0.0154	86.5	0.994	0.00276	91.6	0.983	0.227	25.59	0.966	3.02	0.523
*CoFe*_2_ *O*_4_ *+C*	Cd(II)	77.3 ± 3.8	0.0143	78.1	0.997	0.00273	83.1	0.988	0.233	13.27	0.970	2.71	0.573

**Table 5:** Langmuir and Freundlich models parameters obtained for adsorption of Pb(II) and Cd(II) onto magnetic adsorbents.

Adsorbent	Adsorbate	q_max exp_	Langmuir Model			Freundlich Model		
			q_max_	K	R	K_f_	n	R
		(mg g^−1^)	(mg g^−1^)	(L mg g^−1^)				
*C*	Pb(II)	988.59 ± 49.52	1202.41	0.003207	0.9934	2.6726	1.6974	0.9880
*Fe_3_O_4_+C*	Pb(II)	201.06 ± 12.01	317.60	0.001028	0.9897	54.480	2.4689	0.9669
*CoFe_2_O_4_+C*	Pb(II)	106.09 ± 8.05	122.47	0.002911	0.9869	7.647	2.8496	0.9759
*C*	Cd(II)	165.06 ± 6.60	272.77	0.002468	0.9971	4.7957	1.8078	0.9918
*Fe_3_O_4_+C*	Cd(II)	120.79 ± 6.03	239.88	0.001550	0.9963	1.6506	1.5021	0.9938
*CoFe_2_O_4_+C*	Cd(II)	115.02 ± 6.90	296.06	0.009360	0.9997	0.9266	1.3482	0.9997

**Table 6:** Comparison of adsorption capacities of Pb(II) and Cd(II) on magnetic adsorbents at pH 6.

Precursor	q_max_ (mg g^−1^)				Reference
	Pb(II) 1st Cycle	Cd(II) 1st Cycle	Pb(II) Last Cycle	Cd(II) Last Cycle	
Argan shells (*Fe*_3_*O*_4_*+C*)	389.5	269.0	161.6	252.0	Present study
Argan shells (*CoFe*_2_*O*_4_*+C*)	248.6	264.4	159.7	155.4	Present study
Oil-tea and camellia	225.0	---	211.0	---	[46]
Fresh paulownia tree litter sludge	263.6	---	---	---	[47]
Sludge	206.5	---	165.2	---	[48]
Palm fiber	---	197.96	---	161.6	[49]
Rice straw	---	10.7	---	---	[50]
Palm fiber	188.18	---	150.5	---	[51]
Cane	51.7	--	--	--	[52]
Rice husk	367.6	---	---	---	[53]
Agricultural wastes	229.9				[1]

## Data Availability

Not applicable.

## Supplementary Materials

The following are available online at https://www.mdpi.com/article/10.3390/ma14206134/s1. Figure S1: (a) Nitrogen isotherms at −196 °C, adsorption-open symbols; desorption-closed symbols. (b) BJH pore size distribution obtained from N_2_ desorption isotherms. Samples: ■ *Fe*_3_*O*_4_*+C*, and ▲ *CoFe*_2_*O*_4_*+C*. Figure S2: TEM images. Samples: (a) *Fe_3_O_4_+C*, and (b) *CoFe_2_O_4_+C.* Figure S3: TGA curves in air. Samples: ■ *Fe*_3_*O*_4_*+C*, and ▲ *CoFe*_2_*O*_4_*+C*. Figure S4: Zeta potentials of ■ *Fe*_3_*O*_4_*+C*, and ▲ *CoFe*_2_*O*_4_*+C* as a function of pH. Table S1: Analysis of variance (ANOVA). Factor: Contact time. Table S2: Analysis of variance (ANOVA). Factor: Initial concentration of metal. Table S3: Analysis of variance (ANOVA). Factor: pH. Table S4: Analysis of variance (ANOVA). Factor: reuse cycle.
