# Supplementary material for: Synthesis of Magnetic Adsorbents Based Carbon Highly Efficient and Stable for Use in the Removal of Pb(II) and Cd(II) in Aqueous Solution
Source: Materials (Basel). 2021 Oct 15;14(20):6134. doi: 10.3390/ma14206134 (PMC8539804; doi:10.3390/ma14206134)
Supplement: Supplementary file 1 [file materials-14-06134-s001.zip › materials-1361872-supplementary.pdf]

Article

Synthesis of Magnetic Adsorbents Based Carbon Highly Efficient and Stable for Use in the Removal of Pb(II) and Cd(II) in Aqueous Solution

Safa Benjedim [1], Luis A. Romero-Cano [2,*], Hesham Hamad [1,3], Esther Bailón-García [1], Václav Slovák [4],
Francisco Carrasco-Marín [1] and Agustín F. Pérez-Cadenas [1,*]

[1] Carbon Materials Research Group, Department of Inorganic Chemistry, Faculty of Sciences, University of Granada, Av. Fuente Nueva s/n., 18071 Granada, Spain; safabenj@correo.ugr.es (S.B.); heshamaterials@hotmail.com (H.H.); estherbg@ugr.es (E.B.-G.); fmarin@ugr.es (F.C.-M.)
[2] Grupo de Investigación en Materiales y Fenómenos de Superficie, Departamento de Ciencias Biotecnológicas y Ambientales, Universidad Autónoma de Guadalajara, Av. Patria 1201, 45129 Zapopan, Mexico
[3] Fabrication Technology Research Department, Advanced Technology and New Materials Research Institute (ATNMRI), City of Scientific Research and Technology Application (SRTA-City), Alexandria 21934, Egypt
[4] Department of Chemistry, Faculty of Science, University of Ostrava, 30, dubna 22, 702 00 Ostrava, Czech Republic; vaclav.slovak@osu.cz
* Correspondence: luis.cano@edu.uag.mx (L.A.R.-C.); afperez@ugr.es (A.F.P.-C.)

Citation: Benjedim, S.; Romero-Cano, L.A.; Hamad, H.; Bailón-García, E.; Slovák, V.; Carrasco-Marín, F.; Pérez-Cadenas, A.F. Synthesis of Magnetic Adsorbents Based Carbon Highly Efficient and Stables for Its Use in the Removal of Pb(II) and Cd(II) in Aqueous Solution. *Materials* **2021**, *14*, 6134. https://doi.org/10.3390/ma14206134

Academic Editor: Pasquale Fernando Fulvio

Received: 15 August 2021
Accepted: 12 October 2021
Published: date

Publisher's Note: MDPI stays neutral with regard to jurisdictional claims in published maps and institutional affiliations.

Copyright: © 2021 by the authors. Submitted for possible open access publication under the terms and conditions of the Creative Commons Attribution (CC BY) license (http://creativecommons.org/licenses/by/4.0/).

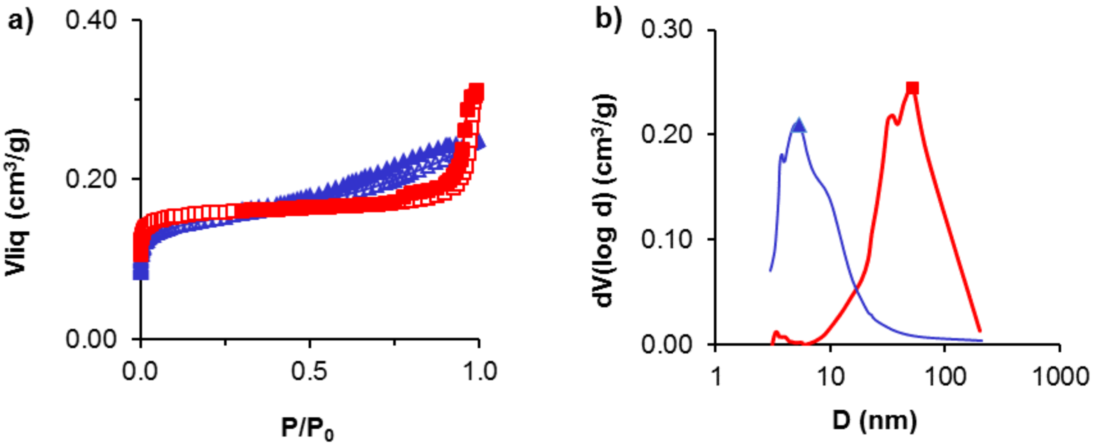

Figure S1. a) Nitrogen isotherms at -196 °C, adsorption-open symbols; desorption-closed symbols. (b) BJH pore size distribution obtained from N_2 desorption isotherms. Samples: ■ *Fe_3O_4+C*, and ▲ *$CoFe_2O_4$+C*.

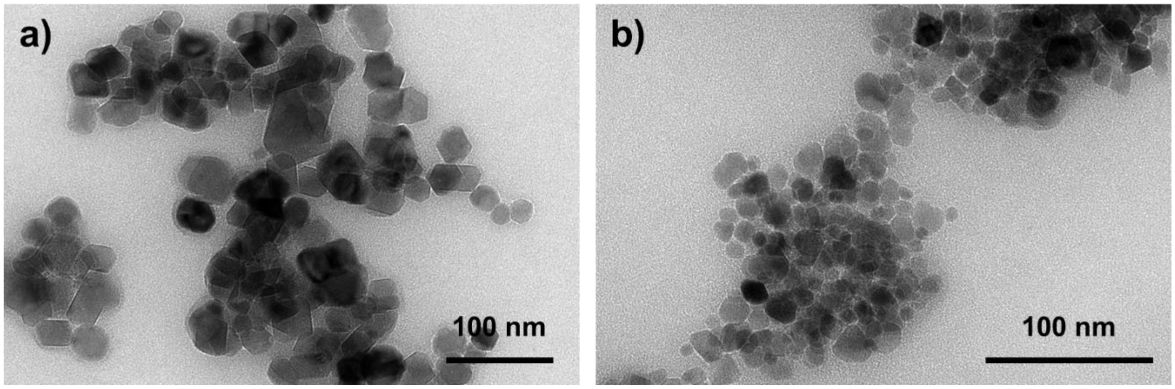

Figure S2. TEM images. Samples: a) *Fe_3O_4+C*, and c) *$CoFe_2O_4$+C*

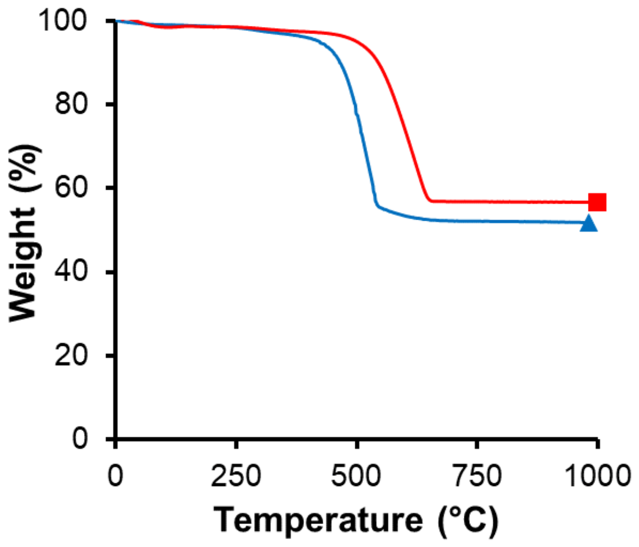

Figure S3. TGA curves in air. Samples: ■ *Fe_3O_4+C*, and ▲ *$CoFe_2O_4$+C.*

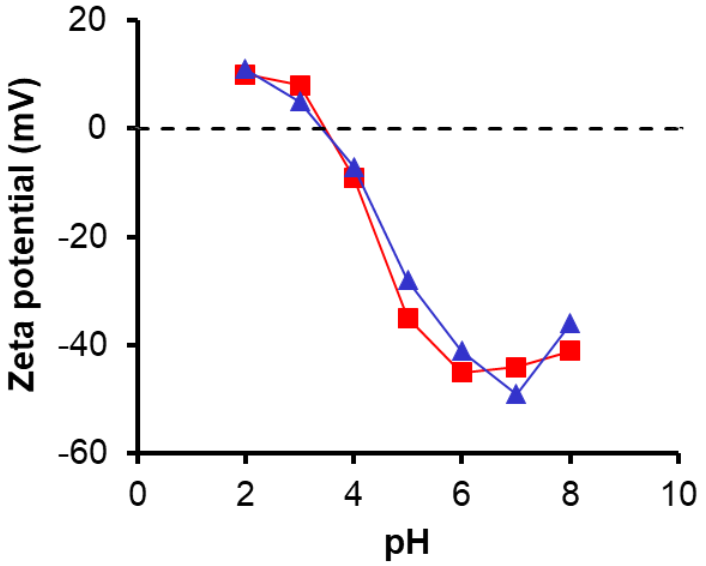

Figure S4. Zeta potentials of ■ *Fe_3O_4+C*, and ▲ *$CoFe_2O_4$+C* as a function of pH

Table S1. Analysis of variance (ANOVA). Factor: Contact time

Adsorption of Pb(II) onto *C*

Effect	SS	Degr. of Freedom	MS	F	p
Intercept	539349.2	1	539349.2	8256.372	0.000000
Contact time	119520.9	5	23904.2	365.926	0.000000
Error	392.0	6	65.3		

Adsorption of Pb(II) onto Fe_3O_4+C

Effect	SS	Degr. of Freedom	MS	F	p
Intercept	1504960	1	1504960	8370.938	0.000000
Contact time	308341	5	61668	343.012	0.000000
Error	1079	6	180		

Adsorption of Pb(II) onto $CoFe_2O_4$+C.

Effect	SS	Degr. of Freedom	MS	F	p
Intercept	374499.9	1	374499.9	6563.955	0.000000
Contact time	105039.0	4	26259.7	460.261	0.000001
Error	285.3	5	57.1		

Adsorption of Cd(II) onto C

Effect	SS	Degr. of Freedom	MS	F	p
Intercept	37169.75	1	37169.75	6437.876	0.000000
Contact time	11357.40	4	2839.35	491.781	0.000001
Error	28.87	5	5.77		

Adsorption of Cd(II) onto Fe_3O_4+C

Effect	SS	Degr. of Freedom	MS	F	p
Intercept	172134.4	1	172134.4	6567.728	0.000000
Contact time	48153.3	4	12038.3	459.318	0.000001
Error	131.0	5	26.2		

Adsorption of Cd(II) onto $CoFe_2O_4$+C.

Effect	SS	Degr. of Freedom	MS	F	p
Intercept	29512.06	1	29512.06	6415.316	0.000000
Contact time	9153.05	4	2288.26	497.421	0.000001
Error	23.00	5	4.60		

Table S2. Analysis of variance (ANOVA). Factor: Initial concentration of metal

Adsorption of Pb(II) onto *C*

Effect	SS	Degr. of Freedom	MS	F	p
Intercept	11532816	1	11532816	21736.59	0.000000
metal concentrations	2774110	15	184941	348.57	0.000000
Error	7428	14	531		

Adsorption of Pb(II) onto Fe_3O_4+*C*

Effect	SS	Degr. of Freedom	MS	F	p
Intercept	547677.1	1	547677.1	14814.60	0.000000
metal concentrations	213985.5	11	19453.2	526.21	0.000000
Error	443.6	12	37.0		

Adsorption of Pb(II) onto $CoFe_2O_4$+*C*.

Effect	SS	Degr. of Freedom	MS	F	p
Intercept	104500.7	1	104500.7	12774.28	0.000000
metal concentrations	22682.9	8	2835.4	346.60	0.000000
Error	73.6	9	8.2		

Adsorption of Cd(II) onto *C*

Effect	SS	Degr. of Freedom	MS	F	p
Intercept	185308.7	1	185308.7	13819.63	0.000000
metal concentrations	83094.9	11	7554.1	563.36	0.000000
Error	147.5	11	13.4		

Adsorption of Cd(II) onto Fe_3O_4+*C*

Effect	SS	Degr. of Freedom	MS	F	p
Intercept	126564.0	1	126564.0	11053.63	0.000000
metal concentrations	44060.4	9	4895.6	427.56	0.000000
Error	114.5	10	11.4		

Adsorption of Cd(II) onto $CoFe_2O_4$+*C*.

Effect	SS	Degr. of Freedom	MS	F	p
Intercept	43440.33	1	43440.33	6683.128	0.000000
metal concentrations	18020.67	5	3604.13	554.482	0.000000
Error	39.00	6	6.50		

Table S3. Analysis of variance (ANOVA). Factor: pH

Adsorption of Pb(II) onto *C*

Effect	SS	Degr. of Freedom	MS	F	p
Intercept	1132740	1	1132740	6356.205	0.000000
pH	65545	3	21848	122.598	0.000216
Error	713	4	178		

Adsorption of Pb(II) onto *Fe_3O_4+C*

Effect	SS	Degr. of Freedom	MS	F	p
Intercept	1077730	1	1077730	5427.324	0.000000
pH	257487	3	85829	432.225	0.000018
Error	794	4	199		

Adsorption of Pb(II) onto *$CoFe_2O_4$+C*.

Effect	SS	Degr. of Freedom	MS	F	p
Intercept	1029613	1	1029613	5385.355	0.000000
pH	255932	3	85311	446.215	0.000017
Error	765	4	191		

Adsorption of Cd(II) onto *C*

Effect	SS	Degr. of Freedom	MS	F	p
Intercept	403637.0	1	403637.0	6216.254	0.000000
pH	32969.2	3	10989.7	169.249	0.000114
Error	259.7	4	64.9		

Adsorption of Cd(II) onto *Fe_3O_4+C*

Effect	SS	Degr. of Freedom	MS	F	p
Intercept	392456.0	1	392456.0	5512.909	0.000000
pH	86215.8	3	28738.6	403.697	0.000020
Error	284.8	4	71.2		

Adsorption of Cd(II) onto *$CoFe_2O_4$+C*.

Effect	SS	Degr. of Freedom	MS	F	p
Intercept	361887.8	1	361887.8	5459.882	0.000000
pH	83787.3	3	27929.1	421.373	0.000019
Error	265.1	4	66.3		

Table S4. Analysis of variance (ANOVA). Factor: reuse cycle

Adsorption of Pb(II) onto *C*

Effect	SS	Degr. of Freedom	MS	F	p
Intercept	349854.3	1	349854.3	4528.536	0.000000
cycle	169611.6	3	56537.2	731.821	0.000006
Error	309.0	4	77.3		

Adsorption of Pb(II) onto Fe_3O_4+C

Effect	SS	Degr. of Freedom	MS	F	p
Intercept	432081.1	1	432081.1	5728.037	0.000000
cycle	75128.1	3	25042.7	331.988	0.000030
Error	301.7	4	75.4		

Adsorption of Pb(II) onto $CoFe_2O_4$+C.

Effect	SS	Degr. of Freedom	MS	F	p
Intercept	297042.6	1	297042.6	6491.252	0.000000
cycle	10650.7	3	3550.2	77.583	0.000532
Error	183.0	4	45.8		

Adsorption of Cd(II) onto *C*

Effect	SS	Degr. of Freedom	MS	F	p
Intercept	565580.5	1	565580.5	6721.743	0.000000
cycle	189.9	3	63.3	0.752	0.575537
Error	336.6	4	84.1		

Adsorption of Cd(II) onto Fe_3O_4+C

Effect	SS	Degr. of Freedom	MS	F	p
Intercept	588282.7	1	588282.7	6723.288	0.000000
cycle	62.3	3	20.8	0.237	0.866447
Error	350.0	4	87.5		

Adsorption of Cd(II) onto $CoFe_2O_4$+C.

Effect	SS	Degr. of Freedom	MS	F	p
Intercept	453659.1	1	453659.1	6480.564	0.000000
cycle	17041.3	3	5680.4	81.145	0.000487
Error	280.0	4	70.0		
